# Late Brain Oligometastases Diagnosed at Least 36 Months after Cancer Detection are Associated with Favorable Survival Outcome

**DOI:** 10.7759/cureus.6553

**Published:** 2020-01-03

**Authors:** Carsten Nieder, Bård Mannsåker, Rosalba Yobuta

**Affiliations:** 1 Oncology, Nordland Hospital Trust, Bodø, NOR

**Keywords:** oligometastases, brain metastases, neurosurgery, long-term survival, radio-surgery

## Abstract

Objective

The aim of this study was to investigate the impact of a long disease-free interval (at least 36 months from the first diagnosis of cancer) on survival in patients with brain oligometastases (maximum four lesions, no extracranial metastases).

Methods

This study involves a retrospective analysis in a group of 89 patients treated with different brain-directed approaches.

Results

We identified seven patients (8%) with an interval from cancer diagnosis to the development of brain metastases of at least 36 months. The median time interval was five months. The one-year survival rates were 80% (interval of at least 36 months) and 43% (shorter interval), respectively (*p *= 0.049). Correspondingly, a large difference in actuarial median survival was observed (39.9 months [95% confidence interval, 16.8-63.0 months] versus 9.7 months (95% confidence interval, 6.1-13.3 months). However, the two Kaplan-Meier curves were not statistically significantly different, *p *= 0.13. In addition to treatment-related differences, the two groups also differed with regard to the type of primary tumor (high versus low rates of non-small cell lung cancer) and gender.

Conclusion

Late dissemination was uncommon. The often applied strategy of effective local treatment for patients with brain-only oligometastases is warranted, especially if the disease-free interval had been at least 36 months. Larger studies are needed to fully understand the impact of confounding factors, such as gender and tumor biology.

## Introduction

Only a small proportion of patients with brain metastases are diagnosed with brain-only oligometastatic disease [1-3]. Commonly, the development of extracranial metastatic sites precedes the development of brain metastases, or widespread intra- and extracranial metastases are detected simultaneously [4-6]. Paralleling the evolving treatment paradigms for liver and lung oligometastases, management of brain oligometastases has changed, too [7-14]. Increasing utilization of surgical resection and/or stereotactic high-dose radiotherapy has resulted in high rates of local control, which in turn has improved overall survival in patients with true oligometastatic disease [1,13]. The fact that a significant proportion of patients are diagnosed with additional metastases within one year of local ablation illustrates the current challenges of diagnostic imaging (presence of small metastases below the threshold of detection), but also tumor biology and genetics, as comprehensively reviewed in references [15-17]. Recently, evidence for heterogeneous metastatic virulence has been provided [18]. As proposed by the authors, it is not sufficient to stratify solely on the basis of the number of lesions and involved sites. Instead, assessing the pace of dissemination is necessary. In a report by Pastorino et al., five-year overall survival was 45% for patients with a disease-free interval of at least 36 months [19]. Treatment consisted of lung metastasectomy in more than 5000 cases. In light of this scientific background, we chose to study the pace of dissemination in a different context, i.e. brain oligometastases. We chose the identical cut-off of at least 36 months of disease-free interval as in reference [19]. We hypothesized that slow dissemination (disease-free interval at least 36 months) would be associated with longer survival also in this patient population.

## Materials and methods

Patients and treatment

We performed a retrospective study of all patients with maximum four parenchymal brain metastases from histologically confirmed extracranial primary tumors treated at our hospital, where we maintain and update a previously utilized database [3,5]. Patients with leptomeningeal metastases or extracranial distant metastases were excluded, resulting in 89 patients with brain-only oligometastases. Their treatment was individualized and consisted of stereotactic radiosurgery (SRS), surgical resection, whole-brain radiotherapy (WBRT), or combinations of different brain-directed approaches. The typical WBRT regimen was 10 fractions of 3 Gy. Systemic treatment was usually prescribed as judged appropriate by the patients’ medical oncologists. The patients were treated between January 01, 2007 and December 31, 2018. Brain magnetic resonance imaging (MRI) was performed to assess the number of intracranial metastases and to exclude leptomeningeal disease. Extracranial staging was not standardized and did therefore not always include positron emission tomography (PET) combined with computed tomography (CT). 

Statistical methods

Overall survival (time to death) from the first day of brain-directed therapy was calculated using the Kaplan-Meier method, and different groups were compared using the log-rank test (SPSS 25, IBM Corp., Armonk, NY, USA). Sixteen patients were censored after a median follow-up of 15 months (range: 2-96 months). The date of death was known in all other patients. Given that the median follow-up was 15 months, we selected the one-year survival rates as the primary endpoint and compared these rates with the two-tailed Fisher's exact probability test (dichotomized by an interval less than 36 versus at least 36 months). Statistical significance was defined as *p *< 0.05 throughout this study.

## Results

Patient characteristics

We identified seven patients (8%) with interval from cancer diagnosis to development of brain metastases of at least 36 months. In 37 patients (42%) brain metastases were detected already at first staging, i.e. simultaneous presentation when diagnosed with cancer. Overall, the median time interval was five months. Further patient characteristics are shown in Table 1.

**Table 1:** Patient characteristics and primary treatment of brain metastases KPS: Karnofsky performance status, NSCLC: non-small cell lung cancer, WBRT: whole-brain radiotherapy, SRS: stereotactic radiosurgery, RT: radiotherapy

	Short interval <36 months	Interval at least 36 months
Median age (years), range	65, 28-90	74, 56-83
Median number of metastases	1, 1-4	1, 1-4
Median KPS	80, 50-100	80, 70-90
Female gender	38 (46%)	7 (100%)
NSCLC	51 (62%)	1 (14%)
Breast cancer	4 (5%)	2 (29%)
Malignant melanoma	4 (5%)	2 (29%)
Kidney cancer	4 (5%)	1 (14%)
Colorectal cancer	6 (7%)	1 (14%)
Others	13 (16%)	0
Neurosurgical resection	32 (39%)	5 (71%)
Primary WBRT	31 (38%)	0
SRS and other RT	19 (23%)	2 (29%)

Survival

**Figure 1:**
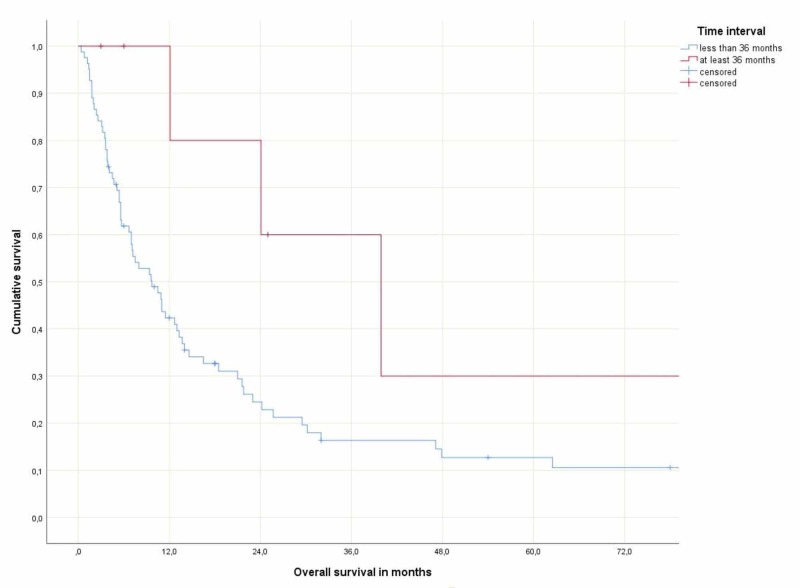
Actuarial overall survival

The one-year survival rates were 80% in patients with a time interval of at least 36 months and 43% (shorter interval), respectively (*p *= 0.049). Correspondingly, a large difference in actuarial median survival was observed (39.9 months (95% confidence interval 16.8-63.0 months) versus 9.7 months (95% confidence interval 6.1-13.3 months). However, the two Kaplan-Meier curves were not statistically significantly different, p=0.13 (Figure 1). 

## Discussion

In a large study of lung metastasectomy by Pastorino et al., five-year overall survival was 45% for patients with a disease-free interval of at least 36 months [19]. In the present study of patients with brain-only oligometastases (less than five), we also found that slow dissemination (disease-free interval at least 36 months) was associated with longer survival. The five-year rate was 30%. When including censored observations with relatively short follow-up of less than one year, the Kaplan-Meier curves did not show a statistically significant difference, despite large differences in actuarial median survival. Unfortunately, the statistical power of a subgroup analysis that only includes seven patients is very limited. We were surprised to discover that very few patients (8%) had time intervals of at least 36 months. On the other hand, previous studies have often reported median time intervals of 12 months or less, indirectly confirming that late dissemination is uncommon [20-25].

Based on the different treatment approaches displayed in Table 1, one cannot exclude that high rates of neurosurgical resection (71%) have contributed to prolonged survival in patients with long time intervals. It is understandable that histological confirmation of brain dissemination is desirable if no extracranial disease activity can be detected and the previous cancer diagnosis was established several years ago. In addition to treatment-related differences, the two groups also differed with regard to the type of primary tumor (high versus low rates of non-small cell lung cancer) and gender. It is thus difficult to conclude that time interval by itself is the main explanation for the favorable survival outcome of patients treated for brain metastases at least 36 months after their initial cancer diagnosis. Unfortunately, the potential role of the confounding factors mentioned above cannot be addressed in a meaningful way with the statistical power provided by the group sizes in the present study. Our findings should stimulate further research, which may provide reliable explanations for the observed differences in survival. In order to create large databases, multi-institutional and international cooperation should be attempted. Clearly, both tumor biology and treatment have the potential to influence prognosis. Despite the lack of definitive answers, our data suggest that the commonly applied strategy of effective local treatment for patients with brain-only oligometastases is warranted, especially if the disease-free interval has been at least 36 months. Long-term survival has also been observed in patients with simultaneous brain metastases and those with relatively short intervals, as shown in Figure 1.

Most patients in this study had solitary brain metastases. Commonly, the presence of 1-4 metastases triggers a recommendation towards SRS or resection rather than WBRT [1,4,13]. Therefore this cut-off was applied in our study. On the other hand, SRS is also feasible in many patients with five or more metastases [12]. Optimal patient selection is an area of ongoing research. In parallel, ablative local therapy for extracranial oligometastases continues to evolve and is hoped to contribute to higher rates of long-term survivors [26-30]. Besides suboptimal statistical power and retrospective collection of information, as well as variable staging procedures (some patients with undetected extracranial metastases may have been included), the present study is also limited by the lack of information about the cause of death and development of widespread metastatic disease during follow-up. 

## Conclusions

Late dissemination to the brain only was uncommon (8%). The often applied strategy of effective local treatment for patients with brain-only oligometastases, e.g. SRS or surgery, is warranted, especially if the disease-free interval before the detection of brain metastases has been at least 36 months. Larger studies are needed to confirm statistical significance (log-rank test) and fully understand the impact of confounding factors, such as gender and tumor biology, which may vary greatly with the type of primary tumor. 
